# The World (of Warcraft) through the eyes of an expert

**DOI:** 10.7717/peerj.3783

**Published:** 2017-09-29

**Authors:** Yousri Marzouki, Valériane Dusaucy, Myriam Chanceaux, Sebastiaan Mathôt

**Affiliations:** 1 Department of Social Sciences, Qatar University, Doha, Qatar; 2 Laboratoire de Psychologie Cognitive, Aix-Marseille Université, Marseille, France; 3 Laboratoire de Psychologie et NeuroCognition, Grenoble, France; 4 Department of Experimental Psychology, University of Groningen, Groningen, Netherlands

**Keywords:** Expertise, Eye-movement, Visual saliency, Pupillometry

## Abstract

Negative correlations between pupil size and the tendency to look at salient locations were found in recent studies (e.g., Mathôt et al., 2015). It is hypothesized that this negative correlation might be explained by the mental effort put by participants in the task that leads in return to pupil dilation. Here we present an exploratory study on the effect of expertise on eye-movement behavior. Because there is no available standard tool to evaluate WoW players’ expertise, we built an off-game questionnaire testing players’ knowledge about WoW and acquired skills through completed raids, highest rated battlegrounds, Skill Points, etc. Experts (*N* = 4) and novices (*N* = 4) in the massively multiplayer online role-playing game World of Warcraft (WoW) viewed 24 designed video segments from the game that differ in regards with their content (i.e, informative locations) and visual complexity (i.e, salient locations). Consistent with previous studies, we found a negative correlation between pupil size and the tendency to look at salient locations (experts, *r* =  − .17, *p* < .0001, and novices, *r* =  − .09, *p* < .0001). This correlation has been interpreted in terms of mental effort: People are inherently biased to look at salient locations (sharp corners, bright lights, etc.), but are able (i.e., experts) to overcome this bias if they invest sufficient mental effort. Crucially, we observed that this correlation was stronger for expert WoW players than novice players (*Z* =  − 3.3, *p* = .0011). This suggests that experts learned to improve control over eye-movement behavior by guiding their eyes towards informative, but potentially low-salient areas of the screen. These findings may contribute to our understanding of what makes an expert an expert.

## Introduction

An expert is someone who is much better at a specific task than a non-expert, or novice. However, what makes an expert an expert? This question has intrigued cognitive psychologists for decades. The emerging picture is that experts, compared to novices, excel in many ways within their domain of expertise: their reasoning is more structured (e.g., Greeno, 1978); they exhibit greater mental flexibility (e.g., Johnson & Raab, 2003); they have greater memory capacity (e.g., Chase & Ericsson, 1982: Ericsson & Kintsch, 1995); and they have increased perceptual skills, that is, increased top-down control over their attention, response inhibition and eye movements (e.g., Underwood et al., 2003; Brefczynski-Lewis et al., 2007).

Usually, experts acquire, over a long period, an extensive knowledge that is specific to one domain (Chase, 1983) and organized in abstract categories and meaningful concepts (Chi, Glaser & Rees, 1982). Some authors consider that the processes involved in the reasoning of experts may be exhibited as perceptual skills (e.g., Klein & Hoffman, 1993, and Laukkonen, 2012 for an extensive review) because the flow of their reasoning is shaped by the tasks that are involved in their domain (e.g., Greeno, 1978). For example, Simon & Simon (1978) analyzed core differences between experts and novices in solving kinematics problems. They concluded that expertise influences the knowledge of concepts and the way individuals achieve solutions. Indeed, experts use a “working-forward procedure” with a weak explicit mention of the goals and sub-goals of the solution, whereas novices use a “working-backward procedure” with a more explicit mention of these goals.

Eye tracking is a common technique to investigate strategies used by experts and novices when exploring their environment. In the domain of medical image inspection, Kundel & Nodine (1978) used in a seminal study eye-movement scanning patterns and the dwell time of fixations to show that unlike novices, experts selectively search for abnormal features when scanning X-ray stimuli. Other oculometric indices such as the amplitude of saccades, the number of fixations and the pupillary response were reported to differ between experts and novices from various domains (see Reingold & Sheridan, 2011).

In the now-classic study of Myles-Worsley, Johnston & Simons (1988) expert radiologists and medical students were presented with pictures of chest X-rays. The results showed that experts attended more rapidly and efficiently to abnormal regions in these images. Norman et al. (1989) replicated these results with dermatologists and concluded that perceptual skills evolve over the course of development, resulting in rapid recognition, discrimination, and an efficient way to learn complex tasks and stimuli. For example, in sports, some findings showed that, unlike novices, elite players exhibit few gaze fixations but with longer durations over a larger spatial window in the visual field (e.g., Williams, Davids & Williams, 1999). This result has been considered as evidence that experts use more efficient search strategies than novices by fixating the most informative locations of the visual scene. These findings were also observed outside the lab (*in situ*) with basketball (Vickers, 1996) and tennis players (Singer et al., 1998). In the same vein, Williams et al. (2002) used eye-movement recordings to compare between skilled and novice tennis players at picking up information from an opponent’s postural orientation. Their results revealed that experts fixate on the head, shoulder, trunk, and hip regions of the body more than novice players who fixate more on arm, hand, racket, and ball regions. These studies confirm that experts devote their visual attention strategically to different areas compared to novices. They rely on their extensive knowledge of their visual environment to guide their eye movements purposefully. Further findings indicate that the type of task and the type of expertise domain make it difficult to generalize the search strategies of experts and novices. For instance, it has been shown that experts have a few fixations with longer duration whereas novices showed the opposite pattern, in reading musical score (Drai-Zerbib & Baccino, 2005), and in playing chess (Reingold et al., 2001). In the same vein, authors such as Williams, Singer & Frehlich (2002) observed longer and more numerous fixations with skilled billiards players than novices in a task with increasing difficulty. Williams et al. (2002) concluded that with difficult tasks, information processing requires a major adjustment in cognitive processes since the workload is significantly heavy to saturate the allocation of attentional resources. In summary, many studies have revealed differences between experts and novices in their patterns of eye movements; however, the nature of these differences varies between studies, presumably because experts adjust their eye movements in different ways, depending on their domain of expertise.

Since Koch & Ullman’s (1985) seminal study, the concept of a saliency map that is purely based on bottom-up properties of the visual scene was popularized as a model and a tool to localize salient points in the visual field. According to an early description of the saliency model by Itti, Koch & Niebur (1998), visual input was pre-processed based on the breakdown of the scene into small topographic feature maps (i.e., one map for orientation, one map for color, etc.) that feed into one big saliency map, which in turn codes for local conspicuity (i.e., salience) within the whole visual scene. Moreover, the saliency model has a general architecture that accounts for bottom-up saliency without the intervention of top-down mechanisms to guide attention. Therefore, this model is a good candidate to test the contribution of saliency to experts’ performance in conjunction with their highly structured knowledge. More recently, Lansdale, Underwood & Davies (2010) reported that expert analysts of aerial photographs performed better than untrained viewers in change-detection and location-memory tasks. Overall, they showed that experts are more accurate in both change-detection and location-memory tasks; however, novices preferably fixate salient features in the presented aerial views. The authors interpreted the reliance of novices on saliency as a lack of semantic information in prioritizing the landing points of their fixations in the scene. Hence, such data favor an explanation in terms of the dominance of top-down processing for experts relative to novices. So far, the above-mentioned studies did not involve the pupillary response.

Since Hess & Polt’s study (1964), it is widely acknowledged that pupil size correlates with cognitive processing demands and the amount of mental effort that people invest in a task (Beatty, 1982). This correlation holds across a wide range of tasks, such as mental arithmetic (Ahern & Beatty, 1979), speech comprehension (Zekveld, Kramer & Festen, 2010) working-memory tasks (Kahneman & Beatty, 1966), and visual search (Porter, Troscianko & Gilchrist, 2007). More recently, Szulewski, Roth & Howes (2015) conducted a study to compare changes in cognitive processing demands between novice and expert physicians using a task-evoked pupillary response (TEPR) paradigm. Participants were invited to answer questions related to arithmetic, general knowledge, and clinical emergency medicine following a gradual difficulty. Overall, difficult questions generated for both groups greater TEPRs relative to easy ones. Also, novices require more mental effort to respond to clinical questions which was reflected by greater TEPRs than trained physicians. The authors concluded that the use of TEPRs is an objective measure to capture the effect of cognitive load in novices and experts.

These studies, and many others, show that the more a task is resource demanding, the more the pupil dilates. Therefore, pupil size is a reliable marker of the amount of mental effort invested by participants in a task, and, consequently, of the performance modulation by mental effort. Other studies showed that the efficient adjustment of mental effort by experts when performing their domain-related tasks could be taken as a signature of their performance. The latter was described in different ways. Indeed, when people face a situation where multiple options are available, many strategies take place. One of the used strategies is called “Take-the-first heuristic”, in which people select the first option that spontaneously comes to mind (Boshuizen & Schmidt, 1992). For example, Raab & Johnson (2007) described the high efficiency level for experts as a “Take-the-first heuristic strategy” given the strong association between experts’ knowledge in a particular domain and the immediate environment where the performance is achieved. Consequently, such association generates multiple options to be considered by experts.

As shown above, unlike novices, experts manage to handle a tremendous amount of information flashing in their eyes in a very efficient way. However, does this imply that they rely less on salient cues and features in the scene? The issue of the primacy of stimulus saliency over prior knowledge in the processing of visual information has not been settled yet in the area of expertise. This question was partly motivated by a long-standing debate in reading and eye movements. The so-called immediate cognitive control model (e.g., Reichle et al., 1998) strongly argued in favor of the semantics guidance of visual explorations of the text. In contrast, low-level models stipulate that the visual saliency of letters and words drive our visual exploration during reading (e.g., Reilly & O’Regan, 1998). However, the generalization to other areas of expertise (i.e., other than reading) remains inconclusive. There are therefore two opposing views regarding the importance of top-down (Folk, Remington & Johnston, 1992; Zelinsky, 2008) and bottom-up factors (e.g., Theeuwes et al., 1998) in the guidance of eye movements. However, a third recent and moderate view assumes that goals and experience may modulate our visual exploration and shape to some extent the weight of what is visually salient and what is not (e.g., Awh, Belopolsky & Theeuwes, 2012). In this regard, the mainstream body of research studying expertise is marked by paradigms that were either lacking complex visual configurations while requiring high-level strategic processing (e.g., chessboard game and sports fields); or having complex visual information with relatively limited involvement of high-level strategic processing (e.g., driving or aircraft simulators).

Recently, a study conducted by Mathôt et al. (2015) used the saliency map technique after measuring participants’ pupil size while they made unconstrained eye movements. The participants performed a visual-search task with different instructions when viewing photographs of natural scene. The authors found a negative correlation between the saliency of locations that were looked at, and the size of the pupil; that is, people were more likely to look at low-salient locations if their pupil was large. The authors interpreted this in terms of mental effort: people would have an automatic tendency to look at salient locations, and mental effort (reflected by a large pupil) would be required to overcome this tendency.

### The present study

In the present study, we focus on how eye-movement behavior in a complex visual task is modulated by expertise. In a relevant review of the literature, Latham, Patston & Tippett (2013) argued that assessing expertise for video game players is not a simple task because it is a multifaceted process that requires besides skills and knowledge, the professional attainment of players as it might reflect more objective measurement of their level of expertise. On the other hand, many studies have shown the boosting effect of video game on players’ cognition and perception. A recent study by Li, Chen & Chen (2016) that showed a causal link between action gaming and the improvement of visuomotor control in video gamers even for 5–6 h of daily practice. The authors invited their participants prior to a target-dot-task to play either Mario Kart, using a steering-wheel controller or Roller Coaster Tycoon III, using a mouse and keyboard. The results showed that playing Mario Kart for 5 h improved participants’ visuomotor control skills when performing the target dot task relative to the group of participants who played Roller Coaster Tycoon.

The review performed by Boot, Blakely & Simons in (2011) on 30 papers comparing performance between video gamers vs. non gamers showed the presence of five major shortcomings in recruiting expert participants: 1. Overt recruiting (possible differential demand characteristics); 2. unspecified recruiting method; 3. potential third-variable/directionality problems (cross-sectional design); 4. no test of perceived similarity of tasks and gaming experience; 5. possible differential placebo effects. Our current recruitment method has been the subject of at least shortcomings 2, 3, and 4.

We focus in this study on World of Warcraft (WoW, Blizzard Entertainment), a massively multiplayer online role-playing game that was released in 2004 (http://gigaom.com/2007/06/13/top-ten-most-popular-mmos/) and has become one of the most popular games in its genre (http://uk.ign.com/articles/2013/07/26/world-of-warcraft-down-to-77-million-subscribers). Being aware of these pitfalls and the difficulty of the recruiting procedure, we have developed an online questionnaire in order to capture the WoW required complex multitasking abilities because it involves completing many stages with various and increasingly difficult challenges and quests as players evolve and constantly navigate in its 3D large virtual landscape. These challenges are genuine problem-solving situations that typically require an optimal solution among numerous different paths that can be eventually taken during the game. The only study that we are aware of about WoW, by Whitlock, McLaughlin & Allaire (2012), showed that WoW has demanding attentional characteristics and can even contribute to the improvement of older adults’ cognitive abilities following a specific cognitive intervention design.

One major novelty of our work is addressing the issue of the impact of visual saliency (i.e., a pure bottom-up information) on expert’s cognitive performance using videos with complex dynamic scenes. In addition, tracking eye movements as a function of the time course of video frames provided us with numerous dynamic cues to examine in grained and ecological ways the visual processing strategies from early to late stages.

Because previous research on expertise has yielded equivocal results (see the studies described in the beginning of the introduction), and little to no research has so far been conducted in the context of complex video games (but see Whitlock, McLaughlin & Allaire, 2012), the present study is exploratory in nature. In the discussion and the results, we will focus on the most important outcomes, but for completeness, all manipulations are described in the method section.

## Method

### Participants

Because there is no available standard tool to evaluate WoW players’ expertise, we built an off-game questionnaire (https://docs.google.com/forms/d/1J44t-W6KM3IQlNlSJvi2nuunQ6W7kAqDhQygeZ4h6ak/viewform) testing players’ knowledge about WoW and acquired skills through completed raids, highest rated battlegrounds, Skill Points, etc. The questionnaire was submitted online on the following famous WoW blogs: http://eu.battle.net/wow/fr/forum/, http://www.jeuxonline.fr/ and http://www.millenium.org/. One hundred forty answers were collected but only 61% of them were analyzed due to either the emptiness or the lack of completeness of the participants’ answers. The nonmetric multi-dimensional scaling technique, which is a multivariate clustering technique that allows to visualize groups of individuals or items based on their similarity,^1^ 1Unlike PCA and other factor analysis methods, non-parametric multidimensional scaling method technique makes ordinal assumptions about the data, which is consistent with the nature of the scale used in our questionnaire. Moreover, it allows an easy interpretation of the structure of the data according to few dimensions (i.e., factors).allowed us to include in the final version of the questionnaire 30 items grouped according to the following three dimensions: 1. Temporal characteristics related to the game (duration of frequency of use: monthly, weekly, daily, etc.); 2. general knowledge of the game (crafts, seasonal events, equipment’s strength, etc.); and the knowledge related to the player’s activity (Valor Points, items’ level, guild, raid level, etc.). Once correctly completed, the questionnaire leads to a maximum score of 64 points. The following inclusion criteria were used: experts must have an overall score above 45, whereas novices must have an overall score below 34. Ultimately, eight participants (three females, age ranged from 17 to 27 years) took part in the experiment (four experts vs. four novices). All were recruited on the basis of their WoW questionnaire score and had played the game in the last month prior to the experiment. Being aware of the psychometric limitations of the questionnaire score as an off-game, rather than in-game index, we cross-validated the questionnaire scores with the participants’ answers in a recognition task that was performed after each video (see the Procedure). The WoW questionnaire scores explain 30% of the overall variability in the recognition scores (*r* = .55, *r*^2^ = .30, *p* < .001), which shows that our questionnaire successfully measured WoW expertise. Participants received monetary compensation. The local ethics committee for experimental research in Aix-Marseille Université approved the experiment.

### Apparatus

An EyeLink 1,000 eye tracker (SR Research Ltd.) was used to measure eye position and pupil size. The eye tracker had a sampling rate of 1,000 Hz and used an automatic saccade-detection algorithm based on a velocity threshold of 30°/s and an acceleration threshold of 8,000°/s^2^, which corresponds to the configuration for the EyeLink 1,000 for cognitive research. Stimuli were presented on a ViewSonic P227 monitor with a refresh rate of 100 Hz and a screen size of 1,024 × 768 pixels. Participants were seated at a viewing distance of 80 cm. Stimulus presentation was controlled with Experiment Builder software (SR Research Ltd., Ottawa, Ontario, Canada). Eye movements were recorded from the right eye. Pupillary size was measured as pupillary diameter. The pupillary size data is not calibrated, and the units of pupil measurement will vary with subject setup. Pupillary diameter is an integer number, in arbitrary units in the range of 400–16,000. It is a noise-limited measure, with noise levels of 0.2% of the diameter, which corresponds to a resolution of 0.01 mm for a 5 mm pupil.

### Stimuli

**Figure 1:**
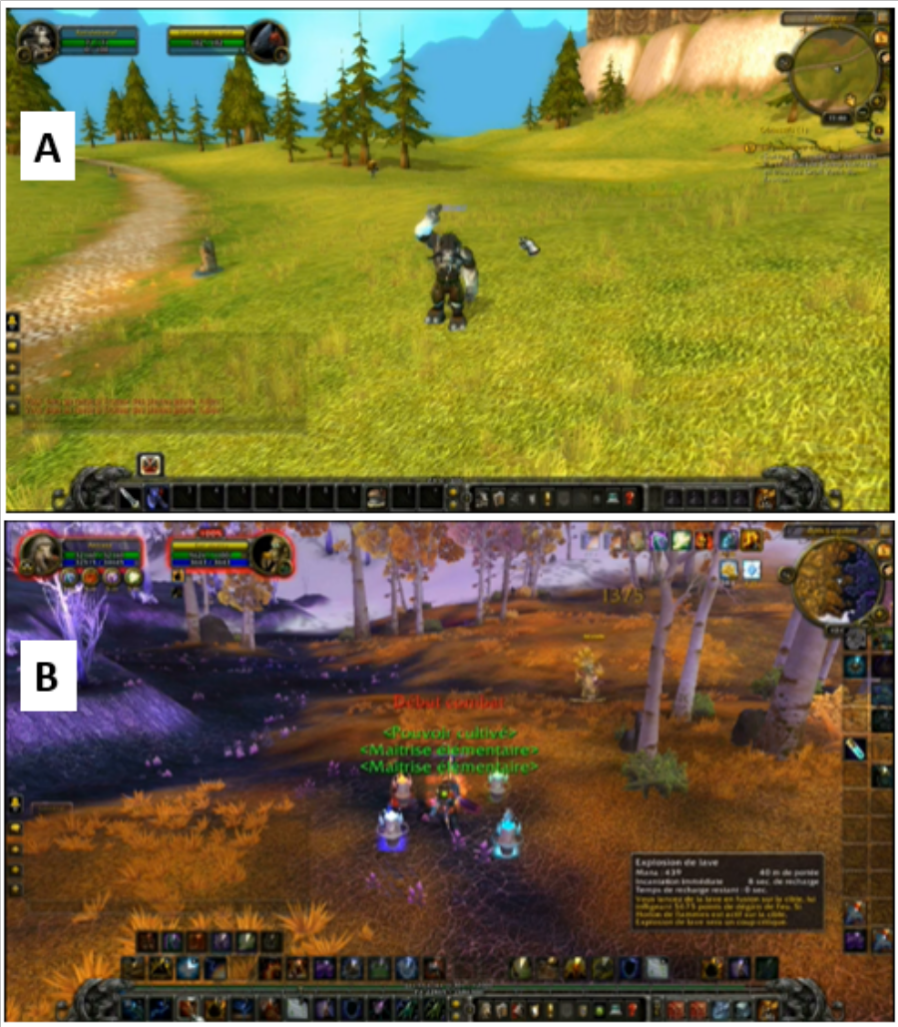
(A) example of a screenshot taken from a simple video consisting of an avatar with less than 10 icons, a simple scenario: “Say hi!” and a simple landscape. (B) example of screenshot taken from a complex video, consisting of an avatar with more than 10 icons, a complex scenario (combat) and a complex landscape. (See also the following online video demo of all the stimuli: https://youtu.be/Kqxm1WkoM5Q.)

For experimental purposes, participants did not play themselves, but viewed in-game videos created by other players. However, all the videos that served as stimuli must accurately meet the requirement for the game visual universe. For this reason, two players who obtained the title of “Game Master” from Blizzard Entertainment within the French WoW players’ community volunteered to create the experimental 24 videos (12 with simple scenario vs. 12 with complex scenario) of 15 s each (see examples in Fig. 1). Each video must respect the following sequence of events: 1. During the first 5 s, the avatar moves forward the landscape; 2. during the next 5 s, the avatar takes a very specific action; and 3. during the final 5 s, the scenario comes to a conclusion (See the demo here https://youtu.be/Kqxm1WkoM5Q). The complexity of the video is based on three criteria: 1. the content of the scenario. For example, “Say hi!” is considered a simple scenario, because it requires little change. In contrast, combat is considered complex (see Fig. 1), because it requires numerous actions and much peripheral information to attend to; 2. the avatar’s level is considered complex if associated with more than 10 icons and finally, 3. the landscape (vacuous or rich). Additionally, 26 audio messages were created, each lasting 13 s. These were either congruent (i.e., the content matches the scenario of the video) or incongruent (the content is related to past or future events relative to the video storyline). The purpose of presenting audio messages is to imitate real game-play settings where most of WoW players tend to use an in-game voice chat through Voice over Internet Protocol such as Skype, Ventrilo, TeamSpeak, Mumble, etc. Each audio message must contain a name (character or region) and an activity. Here is an example associated with one video item: “*This character is a Mage, he is looking for Talent Points to increase his power. Lonely, he travels from region to region and secretly dreams of becoming Draenor Master*”.

### Experimental design

Four experimental factors have been manipulated in this study as follows: expertise level (expert vs. novice) as a between-subjects factor crossed with three within-subject factors: Task Instruction (menu vs. character), Video Complexity (simple vs. complex) and Audio Message (congruent vs. incongruent).

### Procedure

After filling out a consent form, participants watched 24 videos according to the above factorial design in the same (dimly lit room) lighting conditions. In the menu-focus condition, participants were instructed to pay attention to the menu area of the game display. In the character-focus conditions, participants were instructed to pay attention to the main avatar. Instructions were randomized across trials and participants. After each video, participants were presented with six multiple-choice recognition questions, five of which were related to the video content and one of which was related to the audio content. Each question was followed by four alternatives. A difficult multiple-choice question associated with the same audio message example from the Stimuli section is: “The Mage character dreams of becoming: 1. A Sword Master, 2. A Necromancer, 3. A Draenor Master, 4. A Dream Master”. Whereas an easy multiple-choice question is: “The Mage character travels: 1. With his wife, 2. With his cat, 3. With his raven, 4. Alone”. The content of questions and their difficulty varied in an unpredictable way in order to limit expectation effect that may biases visual explorations toward particular regions in the screen. The experimental session lasted 45 min on average.

### Data analysis approach

Our analyses focus on the correlation between pupil size and the saliency of fixated locations (see Saliency analysis). Mathôt et al. (2015) previously reported that this correlation is reliably negative. That this, they found that people are more likely to look at low-salient locations when their pupil is large. Here we investigate whether this correlation differs between experts and novices. In the current study, participants were invited to view dynamic scenes, thus the potential for pupil measurements is expected to be skewed by the angle of the eye, a factor also referred to as pupil foreshortening error (PFE), which is likely to produce a group-effect in pupil size data driven solely by group differences in gaze locations. Given that the patterns of eye movements in this study are expected to systematically differ across the two groups, we have opted for a correction for PFE in the pupil data in order to avoid spurious regularities (Gagl, Hawelka & Hutzler, 2011; Hayes & Petrov, 2016). For each participant, we minimized the error by solving the following multiple linear regression equation :*pupil*_*size* = *α* + *β*_1_*X* + *β*_2_*Y* + *β*_3_*XY*, with the dependent variable *pupil_size* being the pupil size for a fixation, and *X* and *Y* are the *x* and *y* coordinates for that fixation. Once established, the *corrected*_*pupil*_*size*= *pupil*_*size* − *β*_1_*X* − *β*_2_*Y* − *β*_3_*XY*, for each fixation. To test our hypothesis, we have used a combination of techniques, which are described in the Results section. This analysis is exploratory, and does not consider all factors that were manipulated in the Experimental design section. Given the Bayesian inferential nature of the Linear Mixed effects, we reported the JZS Bayes Factor following Rouder et al.’s (2009) and Rouder & Morey’s (2012) recommendations for each computed *t*-test. The JZS Bayes Factor estimates the evidence for the null hypothesis or the alternative. Specifically, the Bayes factor compares two hypotheses: that the standardized effect size is zero or that the standardized effect size is not zero (Rouder et al., 2009). Values greater than 10 indicate strong evidence for the alternative hypothesis, and values greater than 30 are very strong evidence, while values less than 0.1 indicate strong evidence for the null hypothesis, and values less than 0.033 very strong evidence (Jeffreys, 1961).

## Results

### Saliency analysis

For each frame of the video, a saliency map was generated using the NeuroMorphic vision toolkit (Itti, Koch & Niebur, 1998). We used the default parameters, with the exception of using the fast ‘Int’ visual-cortex model for performance considerations. For each fixation, we obtained a saliency value from the corresponding location in the saliency map (see Video in the following online demo of all the material used in the study: https://youtu.be/Kqxm1WkoM5Q). These saliency values are entered as independent variable in the analysis described below.

### Linear mixed effects analysis

Linear Mixed Effects (LME) (e.g., Baayen, Davidson & Bates, 2008; Bates, 2005) modeling was conducted with the corrected pupillary diameter (cPD) as a dependent variable and Saliency, Expertise (novices vs. experts) and video Complexity as independent variables. The following multiplicative LME model formula was implemented: c*PD ∼ Saliency × Expertise* +* (1—subjects)* +* (1—videos)* (as random effects on the intercept) using the lmerTest package of the R software (The R Development Core Team, 2009). The “subjects” and “videos” terms fit random variation between participants and videos in the experiment given the large size of repeated measures that can be extracted from the video stimuli and the conservative criteria we applied that resulted in the selection of few participants in terms of their expertise.

The analysis showed that Saliency has a marginally significant effect on PD for all videos, *t*(6438) =  − 1.7, *p* = .0849, JZS Bayes Factor = 8.1. Pairwise comparison showed that experts (*M* = 2548.1, *SD* = 699.2) showed a larger cPD than novices (*M* = 2244.0, *SD* = 658.5), *t*(6438) = 17.9, *p* < .0001, Cohen’s *d* = 0.45; but this difference is not reflected as a significant main effect in the LME model, *t*(6438) < 1.96. However, the interaction between Saliency and Expertise was revealed to be highly significant, *t*(6438) = 4.35, *p* < .0001, JZS Bayes Factor = 360.7, Cohen’s *d* = 0.12. Thus, Pupil size varies in a differential way as a function of the levels of saliency with experts versus novice. Along with Video Complexity, the Audio message congruency factor does not interact significantly with the other design’s factors, all *t*(24) < 1.96, all ps > .1.

In an extensive analysis, Mathôt et al. (2015) investigated the relationship between pupil size and saliency, and whether this correlation can be explained by other, confounding variables, notably luminance. Their analysis clearly showed that there is a robust correlation between pupil size and saliency, above and beyond a wide range of factors that were considered in their analysis.

### Correlation analysis between saliency and pupillary diameter

**Figure 2:**
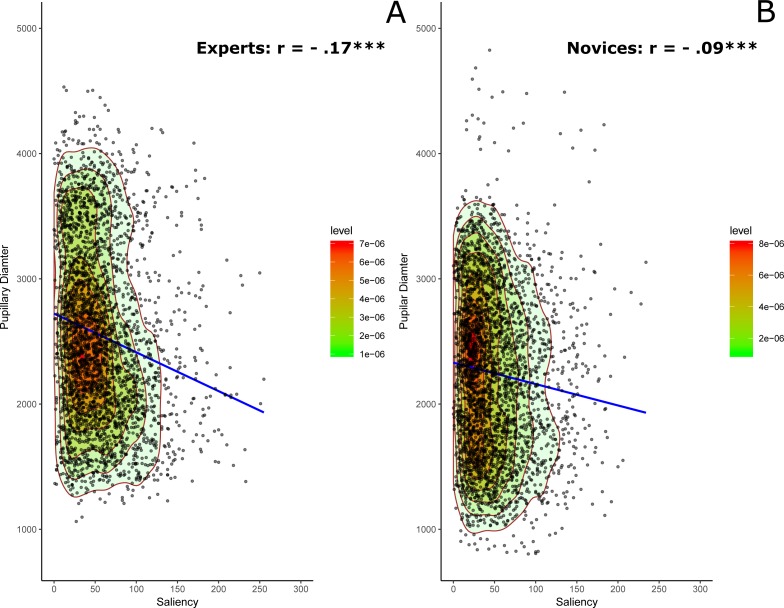
Saliency and pupillary diameter for experts (A) and novices (B). The contours plots generated by the two-dimensional kernel density estimate highlight the shape of the scatter diagram along with regression lines. Colored contours correspond to the boundaries of the sample highest density regions (red region = 25% of the observations).

In this analysis, we tried to examine and compare the shape of the correlations between saliency and the corrected pupillary diameter in both experts and novices. The results revealed a significant negative correlation between saliency and pupillary diameter for both experts, *r* =  − .17, *p* < .0001, Cohen’s *d* =  − 0.34, and novices, *r* =  − .09, *p* < .0001, Cohen’s *d* =  − 0.2. More important, this correlation is significantly stronger with experts than novices as confirmed by the Fisher transformation test, *Z* =  − 3.0, *p* = .0013, Cohen’s *d* =  − 0.12 and by the plots of the probability density function of the data points in the scatter diagram of Fig. 2. The regression analyses revealed a highly significant standardized beta value for the regression slope with experts, *β*_exp_ =  − 1.696e − 01 (*t*_(3195)_ =  − 9.7, *p* < .0001, JZS Bayes Factor = 5.66e+18), relative to novices, *β*_nov_ =  − 9.601e − 02 (*t*_(3241)_ =  − 5.5, *p* < .0001, JZS Bayes Factor = 1.22e+5).

A better description of the data structure can be obtained by using bivariate kernel density estimation (e.g., Duong & Hazelton, 2003) to specify underlying probability density function of the data points. As can be noticed, the saliency distribution in Fig. 2 is considerably left skewed for both groups although experts showed overall stronger correlation compared to novices given the shapes of the colored contours for the density estimation and the steepness of individual the slopes for the regression lines that is more prominent with experts compared to novices.

**Figure 3:**
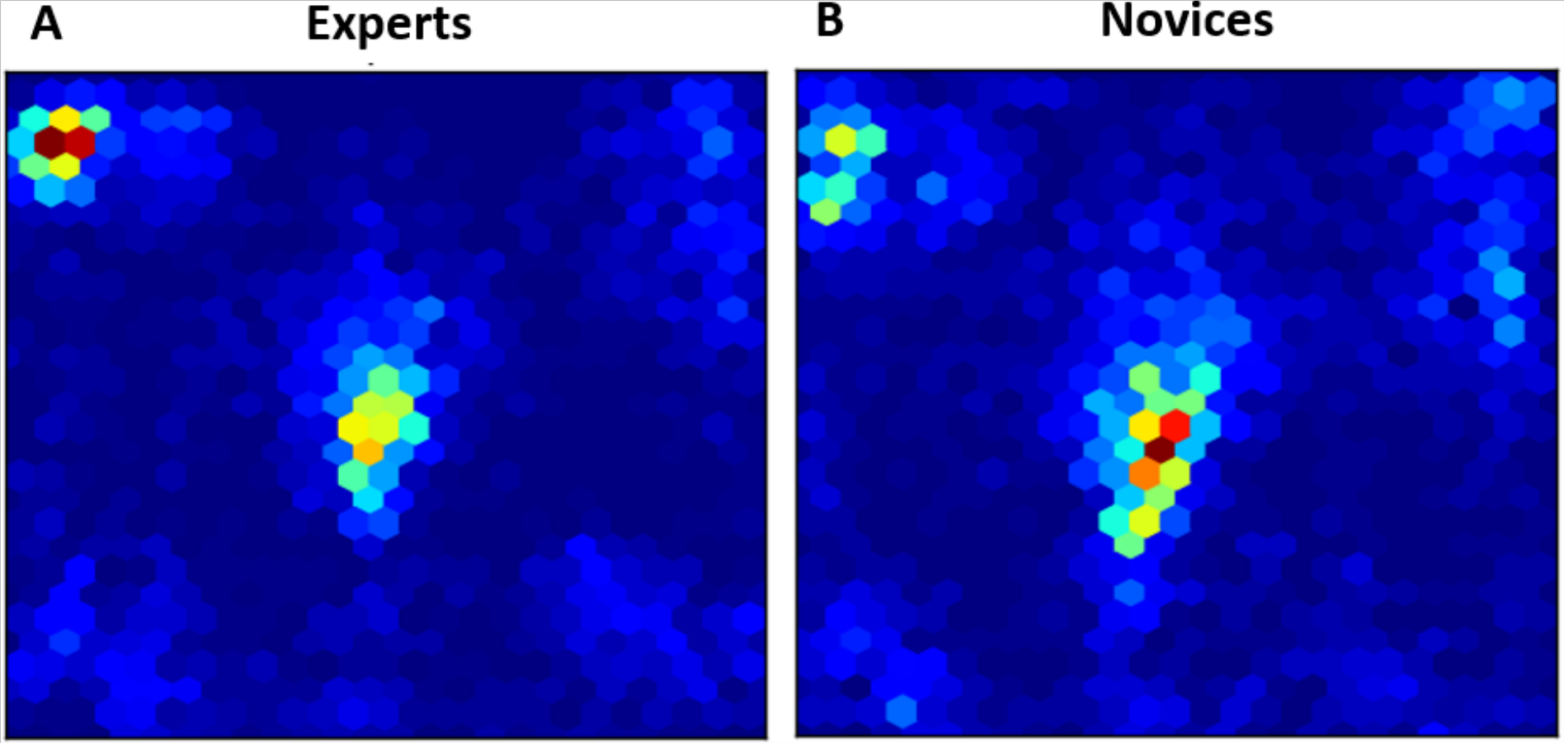
Heatmaps of fixations for experts (A) and novices (B) averaged over all trials.

### The fixations distribution analysis

As mentioned above, eye fixations are used in previous studies as an additional index to compare between experts and novices with respect to their strategies. Hence, we compared in this analysis between the fixation distribution for experts and novices averaged on over all trials. Figure 3 represents two heatmaps for experts and novices with the spatial distribution of all data points relative to their eye fixations as defined by *x* and *y* coordinates on the screen with time being collapsed. According to Fig. 3, experts seemed to be more focused on particular areas than novices and oppositely attributed importance to some spatial regions on the screen.

**Figure 4:**
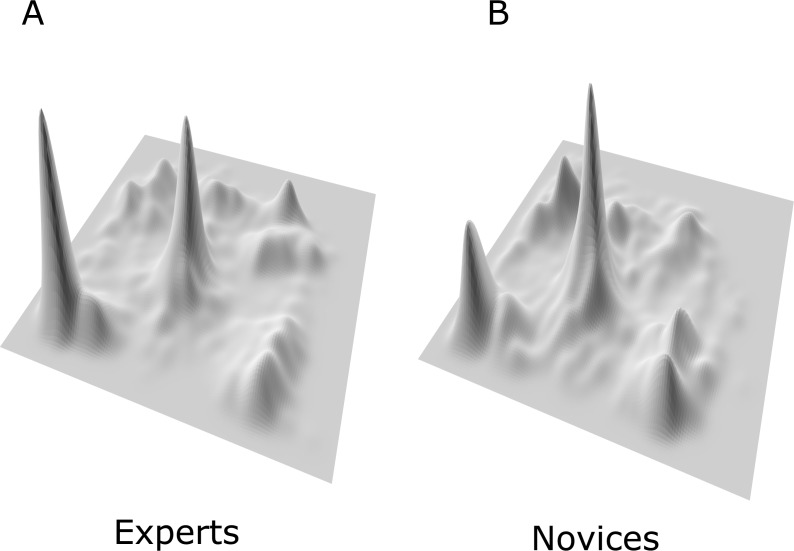
3D representation of the probability density function for experts’ fixation points (perspective plot A) versus novice’s fixation points (perspective plot B).

**Figure 5:**
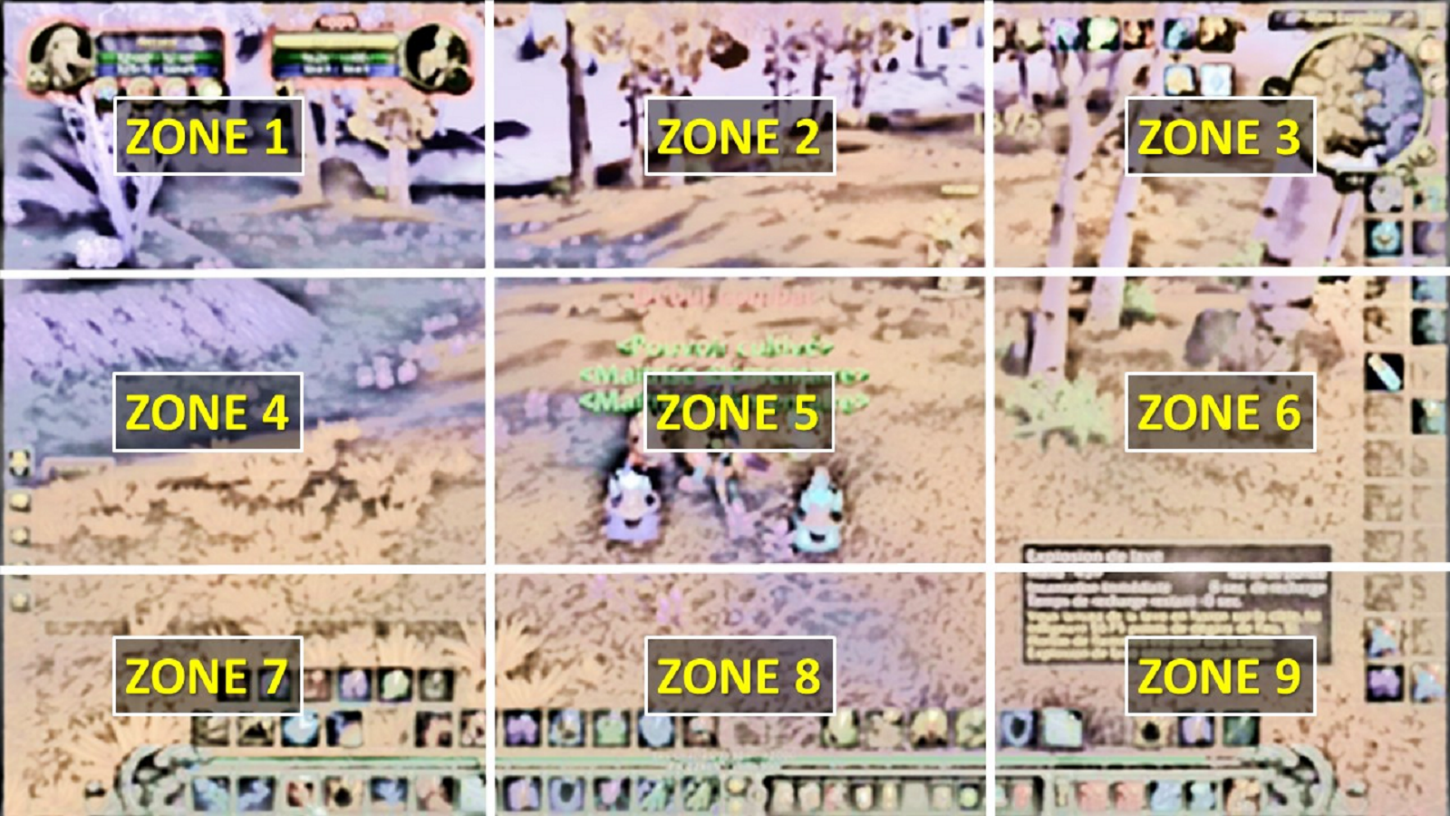
Ad-hoc split of the screens into nine zones reflecting different actions and information content during the video display. The visual angle between the central fixation and the center of zones 4 and 6 is 6.5°; between the central fixation and the center of zones 2 and 8 is 4.8°; and between the central fixation and the center of zones 1, 3, 7 and 9 is 7.2°. Reported visual angles for each zone were based on a viewing distance of 80 cm.

Figure 4 shows two perspective plots of the probability density function (see Castet & Crossland, 2012 for similar use) of the fixation points represented in Fig. 3. As can be noticed, data points for experts and novice have two local modes each also referred to as two loci. The central mode is peaking higher with novices as it reflects their focus on the central target-character zone (Zone 5 in Fig. 5) where most of the actions take place; whereas experts do not lose focus on the character while they pay more attention than novices do to meaningful information (health, combat resources, buffs and rebuffs, etc.) such as the character-player information zone (Zone 1 in Fig. 5).

**Figure 6:**
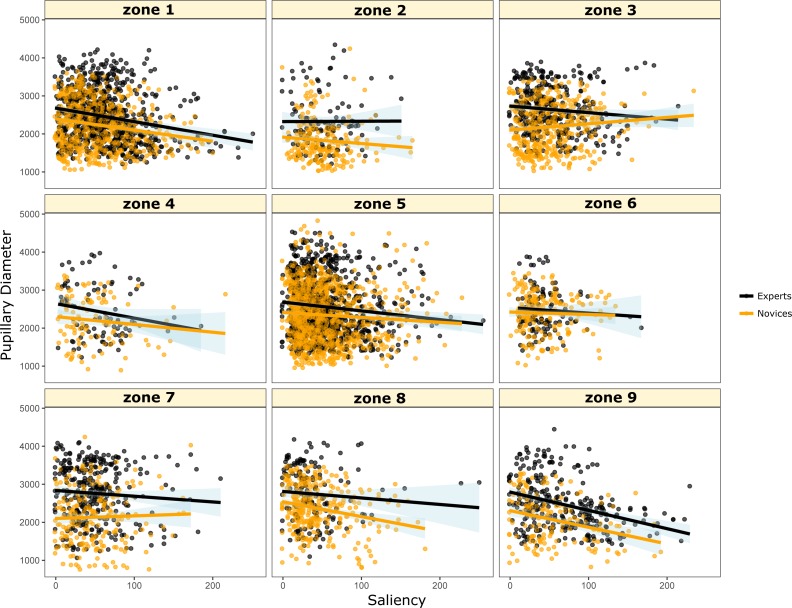
Scatter diagrams and regression lines (plus or minus standard error) of Pupillary Diameter on Saliency as a function of zones of display in the screen and expertise level. Significant differences between these correlations are presented in Table 1.

Based on Fig. 4 loci patterns and the game advisors’ opinion, we divided the display zone into nine sub-zones (see Fig. 5). Although, the zones are unequally distributed in terms of content, action, in-game related information, visual complexity, and the number of eye fixations, the results showed that experts have more dilation in their pupillary diameter compared to novices (all *t*-tests are highly significant, *ps* <  .0001), except for zone 6 where no difference was observed between the two groups (See Fig. 6).

Overall, Fig. 6 and Table 1 show that experts have higher negative correlation between saliency and pupillary diameter with steeper slopes for the regression lines relative to novices. In other words, the higher the visual salience of the video, the smaller the pupil diameter. Moreover, only zone 1 drives the most significant difference between experts and novices. This same result is mirrored in Fig. 4 where experts exhibit higher pick of fixation points in zone 1 compared to novices, which supports the idea that experts rely efficiently on their knowledge and strategies in the game while being able to thwart the noise coming from a huge amount of visual information within a time window that does not exceed 1,000 ms.

### Correct responses analysis

The results of the mixed ANOVA conducted on the percent of correct answers in the recognition task after the video display revealed a significant main effect of Expertise, *F*(1, 6) = 9.7, *MSE* = 1.2, *p* < .02, Cohen’s *d* = 3.1, with experts being more accurate than novice to recall auditory and visual information. Also, experts performed better than novices in this task in the presence of complex video and incongruent messages, *F*(1, 6) = 7.5, *MSE* = 1.5, *p* < .03, Cohen’s *d* = 2.7.

**Table 1:** Statistical comparisons of the correlations between pupil diameter and saliency as a function of the screen zones displayed in Fig. 5 and expertise level.

Zones	Correlations with experts	Correlations with novices	Comparisons and effect size
Zone 1	−0.211 (*n* = 780, *p* < .0001)	−0.078 (*n* = 574, *p* < .05)	*Z* = − 2.47, *p* = .013 Cohen’s *d* = − .07
Zone 2	0.004 (*n* = 123, *p* = .967)	−0.062 (*n* = 219, *p* = .49)	*Z* = 0.58, *p* = .562 Cohen’s *d* = 0.03
Zone 3	−0.108 (*n* = 365, *p* < .05)	0.055 (*n* = 416, *p* = .29)	*Z* = − 2.27, *p* = .112 Cohen’s *d* = 0.08
Zone 4	−0.188 (*n* = 90, *p* = .075)	−0.238 (*n* = 104, *p* < .05)	*Z* = 0.36, *p* = .718 Cohen’s *d* = 0.02
Zone 5	−0.116 (*n* = 940, *p* < .0001)	−0.147 (*n* = 1029, *p* < .0001)	*Z* = 0.7, *p* = .483 Cohen’s *d* = 0.015
Zone 6	−0.061 (*n* = 109, *p* = .522)	0.011 (*n* = 209, *p* = .905)	*Z* = − 0.6, *p* = .548 Cohen’s *d* = − 0.03
Zone 7^a^	−0.230 (*n* = 319, *p* < .0001)	−0.123 (*n* = 223, *p* < .0001)	*Z* = − 1.3, *p* = .207 Cohen’s *d* = − 0.05

**Notes.**

a Zone 7 (also refers in this table to menu zone) encompasses zone 7 + zone 8 + zone 9 as shown in Fig. 5.

## Discussion

The aim of the present study was to examine the visual explorations strategies of experts in a visually complex domain and with dynamic scenes. We chose the massively multiplayer online role-playing game World of Warcraft (WoW) as a new experimental paradigm to explore the strategies used by novices and experts to handle a massive amount of complex visual information while inhibiting stimulus saliency in order to take over control of their visual processing of a scene. Eye movements and pupil size were recorded during participants’ exploration of 24 videos presenting various scenarios of the game during 15 s each.

Our main finding is that there is a negative correlation between pupil size and saliency, and that this correlation is stronger for experts than for novices. The negative correlation between pupil size and saliency has been reported previously (Mathôt et al., 2015) and has been interpreted in terms of mental effort. By default, (i.e., if people do not invest mental effort) the pupil is small (e.g., Beatty, 1982) and salient objects, such as sharp corners and bright lights, predominantly capture the eyes. In contrast, if people invest mental effort to, for example, locate a set of lost keys, the pupils dilate, and the eyes are captured to a lesser extent by saliency. The fact that the relationship between saliency and pupil size appears to be modulated by expertise is intriguing. One possible explanation is that experts have better control over their eye-movement behavior than novices. Following this logic, experts know which parts of the game display are relevant, and, with some effort, they can use this knowledge to guide their eye movements. In contrast, novices do not have this domain-specific knowledge, and are unable to effectively control their eye movements, even when exerting mental effort. This interpretation is in line with Lansdale, Underwood & Davies (2010), who suggested that experts rely more on the semantics of the visual scene (i.e., domain-specific knowledge), relative to novice, who rely more on bottom-up cues. At first sight, this seems to imply that experts should, on average, look at less salient locations than novices. However, this does not necessarily need to be the case, because saliency often coincides with relevance (e.g., Henderson, Malcolm & Schandl, 2009), and experts can make use of this fact. Rather, our data suggest that experts, relative to novices, are better able to prioritize relevance of saliency when they try to do so, leading to an stronger negative correlation between pupil size (as a marker of mental effort) and the tendency to fixate salient locations.

Moreover, the distribution of the fixations for experts and novices seem different (see Fig. 3 and Table 1). Indeed, two main regions were the most fixated during the video display by all participants: the central character zone (zone 5) and the information zone (zone 1). However, it seems that the goals of novices and experts are different, because experts fixate zones that are relevant for the game, more frequently than do novices. Novices, on the other hand, fixate the character zone more than do experts. One tentative explanation is that novices systematically face a lack of knowledge of the environment, which causes them to gather as much information as they can in order to achieve their goal. On the contrary, experts set up a qualitative exploration by focusing on the most relevant information regardless of the size of visual information in the scene as shown in Table 1 results. The latter finding joins a well-established statement according to which the flow of experts’ reasoning is sharpened by the tasks that are typically used to perform in their specific domain (e.g., Greeno, 1978).

We can assume that expert players implicitly learned from previous experience that saliency is informative for the game; they also learned to inhibit parts of visual information in order to adjust their performance while playing the game. Conversely, novices seem to look at less salient things. Familiarity can be also a contributing factor as shown recently by Lansdale, Underwood & Davies (2010), where experts’ compensation saliency influence by retrieving specific landmarks information about particular objects or items in the scene, which may explain the difference in their pupillary diameter, compared to novices (See Fig. 6). This idea is in line with the study of Li, Chen & Chen (2016) that showed a causal link between action gaming and the improvement of visuomotor control in video gamers even with a few hours of practice.

In the same vein, the retrieval of particular salient information could explain why our experts were significantly better than novices even in the presence of complex video or with incongruent audio message to answer more accurately to the recognition test following videos’ display.

These findings are in line with previous studies that showed that novices did not possess flexible strategies while experts are able to extract similarities among various situations using a more abstract and global (holistic) approach to the problem or the situation (Dreyfus & Dreyfus, 1986). Because experts articulate task goals with less effort and fittingly to the situation at hand, they access very rapidly their large organized knowledge in their memory to drive their strategies in problem solving. The higher performance observed with experts in our study corroborates the idea that the special reasoning of experts can be mirrored in their perceptual skills (e.g., Klein & Hoffman, 1993).

One limitation of the current study is the size of the sample. With a large sample, some other factors such as the age of participants may be interesting to look at as a modulatory factor for the expertise. Another limit lies in the fact that experts’ pupil dilation may also be the results of the effect of an increased arousal (e.g., Bradley et al., 2008) when facing test stimuli that reflect familiar situations found in their gaming environment. Hence, it is not excluded that the stronger correlation observed with experts is partly driven by potential confounding influence of arousal or even motivation and expectation to play the game.

In summary, we have shown that there is a negative correlation between pupil size and the saliency of fixated locations, such that people are more likely to look at low-salient locations when their pupil is large. This finding is consistent with a recent study (Mathôt et al., 2015), and is believed to reflect the mental effort that participants invest in eye-movement guidance: when they invest little effort, their pupil is small, and their eyes are mainly drawn to salient parts of a scene. In contrast, when participants invest a lot of effort, their pupil dilates, and their gaze is more often directed at parts of the scene that are relevant to the task at hand, but may not be very salient. Crucially, we have found here that the negative correlation between pupil size and saliency is especially strong for experts. We have interpreted this as the experts’ learning to improve control over eye-movement behavior by guiding their eyes towards informative (e.g., Jarodzka et al., 2010), but potentially low-salient areas of the screen. In line with major models of expertise, experts are able to retrieve quickly larger features of visual information owing to their larger retrieval structures or chunks. These findings extend to the case of video game players that experts use more effective and efficient visual search behaviors and possess greater knowledge of situational information (Goulet, Bard & Fleury, 1989; Williams, Davids & Williams, 1999). The current study helps to further shape our understanding of this characteristic by revealing that experts have developed over time and through learning the ability to control and to filter out more efficiently than novices the flood of visual information in their domain of expertise as evidenced by our pupillometric approach.

##  Supplemental Information

10.7717/peerj.3783/supp-1 Supplemental Information 1 Pupillometric data

10.7717/peerj.3783/supp-2 Supplemental Information 2 Appendix 1/Consent Form

## Additional Information and Declarations

### Data Availability

The following information was supplied regarding data availability:
https://youtu.be/Kqxm1WkoM5Q.
