# Supplementary material for: The World (of Warcraft) through the eyes of an expert
Source: PeerJ. 2017 Sep 29;5:e3783. doi: 10.7717/peerj.3783 (PMC5624293; doi:10.7717/peerj.3783)
Supplement: Supplemental Information 2 [file peerj-05-3783-s002.pdf]

Appendix 1

Expertise in World of Warcraft (WoW) Online Questionnaire

This questionnaire aims to collect information on WoW playing knowledge and techniques. Please answer as honestly as possible; your data will remain fully anonymous.

1. Have you ever played the game Warcraft on PC?
   - Yes
   - No

2. Since when do you play WoW?

3. How many times did you log on WoW in the last 30 days?

4. How many hours per week do you play WoW?

5. How many hours a day do you play WoW?

6. Cite the maximum of seasonal events celebrated in WoW

7. What are the two titles that do not belong to WoW?
   - The explorer
   - Heart of artichoke
   - The joyful
   - Jenkins
   - The lover
   - Ambassador
   - Hand of A'dal
   - Champion of white lands
   - Invincible winner
   - I don't know

8. What are the three professions that do not exist in WoW?
   - Engineering
   - Tailoring
   - First aid
   - Herbalism
   - Jewel crafting
   - Firefighting
   - Inscription
   - I don't know

10. If the name of a character is written in orange, does it necessarily mean that he will attack you?
    - Yes
    - No

11. At what level talents appear in WoW ?

12. What is the main currency used in WoW?

13. The strength of an equipment can be recognized by the color with which it is written. Indicate among these statements the correct order of colors from the lowest to the highest strength.
    - white-gray-blue-green-purple-orange
    - blue-green-orange-gray-white- purple
    - gray-blue-white-orange-green-purple
    - white-gray-purple-green-blue-orange
    - gray-white-blue-green-purple-orange
    - gray-white-green-blue-purple-orange

14. How is called the raid where the Lich King was killed?

15. Taking into account the game upgrades, give the maximum number of allowed players in a raid:

16. On what server are you playing?
- RPPVP
- PVP
- RP
- PVE
- I don't know

17. What is the level of your most experienced avatar?

18. In which area of WoW your most experienced avatar is based?

19. What is the name of the hardest raid you have done or you are about to do?

20. Choose among the following list of item sets one you have acquired recently:
- Dungeon 1
- Dungeon 2
- Dungeon 3
- Raid T1
- Raid T2
- Raid T3
- Raid T4
- Raid T5
- Raid T6
- Raid T7
- Raid T7.5
- Raid T8
- Raid T8.5
- Raid T9
- Raid T9.5
- Raid T9.9
- Raid T10
- Raid T11
- Season 1
- Season 2
- Season 3
- Season 4
- Season 5
- Season 6
- Season 7
- Season 8
- Season 9
- I don't know

21. What is your item level?

22. How many Valor Points have won your most experienced avatar?

23. How many Justice Points have won your most experienced avatar?

24. What are the three main roles a player can embody in WoW?

25. What are the different classes that a player can embody in WoW?
- Shaman
- Warlock
- Mage
- Priest
- Hunter
- Demom Hunter
- Valkyrie
- Thief
- Death Knight
- Dark elf
- I don't know

26. What version of WoW do you play or have you played?
- World of warcraft
- The burning crusade
- Wrath of the Linch king
- Cataclysm
- I don't know

27. Are you a guild member?
- Yes
- No

28. What is the level of the guild in which you are using your most experienced avatar?

29. How many points of epic deeds your guild has?

30. How many points of epic deeds your most experienced avatar has?

Psychometric characteristics of the questionnaire:

85 out of 140 responses were retained. The remaining answers were discarded due to incongruence or incomplete responses. Preliminary results showed a normal distribution of the scores that range from 15 to 45 points. The matrix of the Euclidean distances between items allowed us to remove three items from the original version that deviate the most from the centroid of the matrix. These items concern old events or tricky details about the video game: i) *the corrupted blood incident*; ii) *the Temple of Ahn'Qiraj hard mode*; and iii) *how to reach the Obsidian Sanctum*. Following this step, the non-metric multidimensional scaling technique (non-metric MDS) was applied to categorize the remaining 23 questions in four dimensions:

1. Time spent on playing the game
2. General knowledge about the game
3. General knowledge about the different raids
4. In-game activities

However, the release of the expansion "Cataclysm" during the running of the online questionnaire has triggered new in-game activities. Consequently, we made few adjustments in order to update the first version accordingly by adding new questions about Item levels, Guilds, Valor and Justice Points. The second and final version of the questionnaire (see Appendix 1) was adopted by grouping 30 questions into three dimensions instead of four and with a maximum score of 64 points. The three dimension that give the best-fit are

I. Time spent on playing the game (frequencies were evaluated on a monthly, weekly and daily basis)
II. General knowledge about the game (Skills, Seasonal events, Strength of the equipment, etc.)
III. Knowledge related to in-game activities such as Valor Points, Item levels, Guilds, Raid levels, etc.).

The questionnaire allowed us to define three levels of expertise: Experts score more than 45 points, Intermediate players score between 44 and 35 points, and novices score less than 34 points.
